# Role of quality control circle in sustained improvement of hand hygiene compliance: an observational study in a stomatology hospital in Shandong, China

**DOI:** 10.1186/s13756-016-0160-1

**Published:** 2016-12-08

**Authors:** Peng Chen, Ting Yuan, Qinfeng Sun, Lili Jiang, Hongmin Jiang, Zhenkun Zhu, Zexin Tao, Haiyan Wang, Aiqiang Xu

**Affiliations:** 1 Department of Epidemiology, School of Public Health, Shandong University, Jinan, Shandong People’s Republic of China; 2 Shandong Provincial Key Laboratory of Oral Tissue Regeneration, Shandong University, Jinan, Shandong People’s Republic of China; 3 School of Stomatology, Shandong University, Jinan, Shandong People’s Republic of China; 4 Department of General Medicine, Shandong Provincial Hospital Affiliated to Shandong University, Jinan, Shandong People’s Republic of China; 5 Shandong Provincial Key Laboratory of Infectious Disease Control and Prevention, Jinan, Shandong People’s Republic of China; 6 Shandong Center for Disease Control and Prevention, No. 16992 Jingshi Road, Jinan, 250014 Shandong People’s Republic of China

**Keywords:** Hand hygiene, Quality control circle, Healthcare-associated infection, Healthcare workers

## Abstract

**Background:**

Hand hygiene is an important element of the WHO multimodal strategy for healthcare-associated infection control, whereas compliance of hand hygiene among healthcare workers (HCWs) remains a challenge to sustain. In order to increase the hand hygiene compliance of HCWs, a quality control circle (QCC) program was carried out in our hospital, and the plan-do-check-act (PDCA) method was applied for 12 months.

**Findings:**

Hand hygiene compliance rates improved over time, with significant improvement between preintervention (60.1%) and postintervention (97.2%) periods (*P* < 0.001). Nurses (88.3%) exhibited higher compliance than dentists (87.3%), and female (88.4%) HCWs were more likely to perform hand hygiene than males (85.6%), both *P* < 0.001. Overall hand hygiene compliance and observance of the five indications exhibited significant linear increases over time (*P* < 0.005).

**Conclusion:**

This study highlights the success of a multifaceted intervention, conducted by QCC program and PDCA method, which led to a significant improvement of hand hygiene compliance. Though training is the most basic intervention element, surveillance, evaluation and feedback should be explored as additional interventions to ensure that hand hygiene compliance is achieved and sustained at high levels.

**Electronic supplementary material:**

The online version of this article (doi:10.1186/s13756-016-0160-1) contains supplementary material, which is available to authorized users.

## Background

Quality control circle (QCC), first established in Japan in 1962, has been widely used in medical and healthcare fields in Germany, Austria and Thailand [1–3]. In 2001, QCC was introduced to medical institutions in China, aiming to increase the quality of medical service by improving medical workers’ awareness of spotting and solving medical problems [4–6]. Healthcare-associated infection (HAI) have been identified as a major preventable complication for inpatient care, and hand hygiene was recommended as one of the most effective strategies to prevent HAI [7]. Hand hygiene is defined as any action of hand cleansing, including practices such as antiseptic hand washing, antiseptic handrubbing, hand washing, hygienic handwash, surgical handscrubbing, etc. [8]. However, the compliance of hand hygiene is generally poor despite a variety of measures were proposed to improve it [9–11]. The World Health Organization (WHO) launched the global hand hygiene campaign in 2004 to improve hand hygiene compliance, which included five indications of before patient contact, before an aseptic task, after body fluid exposure risk, after patient contact and after contact with patient surroundings. The evaluation and feedback of hand hygiene performance are important elements of this campaign.

In 2013, a QCC program was carried out in department of implant dentistry in Stomatology Hospital of Shandong University, aiming to improve the hand hygiene compliance of healthcare workers (HCWs) by active monitoring using covert observation. Patients treated in this department were all outpatients with dental implantation treatment, and no intravenous line or other indwelling device was applied. The program was reviewed, revised and promoted monthly. This was the first time that the QCC program was explored for the improvement of hand hygiene compliance in our hospital, and it also promoted the application and practice of advanced tools for the quality management and control.

## Methods

**Fig. 1:**
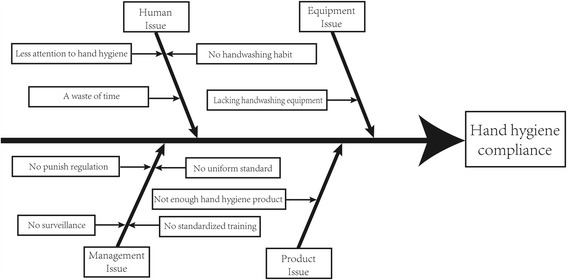
The reasons for low hand hygiene compliance demonstrated in a fishbone diagram

The QCC program was launched based on the QCC theory [12], and the basic steps of QCC program were outlined in Additional file 1. The roles of QCC program included project director, counselor, circle head and circle members, which were acted by infection control officer, charge nurse, department head and the rest dentists and nurses, respectively. The program started with the circle head and members understanding the status quo of hand hygiene compliance and investigating the reasons through brainstorming. A fishbone diagram demonstrated the possible reasons for low hand hygiene compliance in this program (Fig. 1). The QCC program was revised and promoted monthly in accordance with plan-do-check-act (PDCA) cycle. Hand hygiene compliance was summarized and evaluated by the project director, and periodic feedback was implemented to all QCC members at the circle meeting each month. An agenda of the circle meeting was showed in Additional file 2.

Intervention measures were then analyzed and modified through quality management approaches. Firstly, hand hygiene products included an alcohol based hand rub agent and liquid soap were increased to at least one for each dental unit to improve the accessibility. Secondly, establishing a covert observer unit was a necessary component to investigate the hand hygiene compliance by unbiased covert observation. Moreover, circle meeting was the most important form to promote the smooth execution of QCC program and was held at least once a month, with an activity log was maintained. At the QCC meeting, hand hygiene compliance was posted up and red flags were awarded to the exceptional individuals. Also, punishments such as paying a fine would be executed to the noncompliant HCWs to hold the accountable for their practices.

We invited medical students to serve as the covert observers during their clinical clerkships. A standard data form was used to record the six hand hygiene compliance, which included WHO recommended indications [8]. As the failure to change gloves between clinical procedures would increase the risk of cross-transmission [13], the indication of after removing gloves was added in our study. All the data was analyzed with SPSS version 18.0, assuming a type I error of *α* = 0.05. The Chi square test was used to test the statistical difference in hand hygiene compliance by professional category and gender. Cochran Armitage trend test was used to analyze the linear trend of hand hygiene compliance over time, and the processes were illustrated in Additional file 3.

## Results

**Fig. 2:**
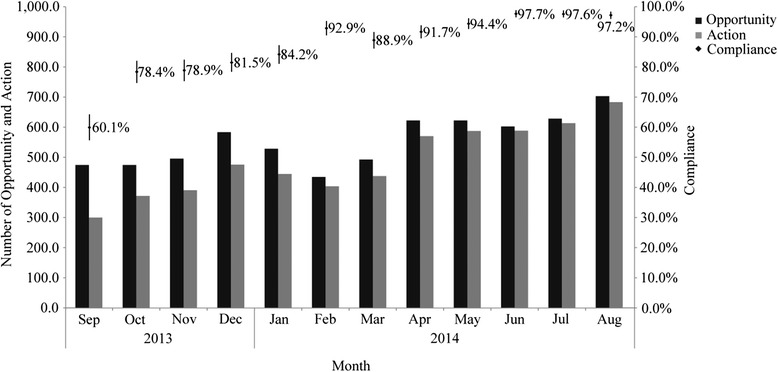
Monthly hand hygiene compliance estimates (95% confidence intervals) and sample sizes

From September 2013 to August 2014, a total of 6681 hand hygiene opportunities were detected by direct observation in department of implant dentistry, and 5869 hand hygiene actions were recorded. Kolmogorov-Smirnov method showed that the data in each month were subject to a normal distribution, and the mean was used to represent the average compliance during the QCC program. The results of Chi square test revealed that nurses (88.3%) exhibited higher hand hygiene compliance than dentists (87.3%), and the female (88.4%) were more likely to perform hand hygiene than the male (85.6%), both *P* < 0.001 (Table 1). Monthly hand hygiene compliance and the respective 95% confidence interval were displayed in Fig. 2. During the QCC program, 436 to 705 hand hygiene opportunities were calculated monthly by the covert observers, with an average of 556.7 per month. The hand hygiene compliance was 60.1% as the QCC program was launched, and eventually the compliance was stabilized with an average rate of 97.5% for the last three months. Cochran Armitage trend test was applied to evaluate the linear trend of hand hygiene compliance, and the result showed that the compliance exhibited a significant linear increase over time (*χ* ^*2*^ = 515.833, *P* < 0.005).

**Table 1:** Descriptive analysis of hand hygiene opportunities and actions among different parameters

Parameter	Opportunity	Action	Compliance	*χ* ^*2*^	*P*
Total	6681	5869	87.8%		
Professional category					
Dentists	3046	2659	87.3%	51.927	<0.001
Nurses	3635	3210	88.3%		
Gender					
Male	1309	1121	85.6%	2470.883	<0.001
Female	5372	4748	88.4%		
Hand hygiene indications^a^					
1. Before patient contact	1410	1079	76.5%	468.466	<0.005
2. Before an aseptic task	991	946	95.5%	24.058	<0.005
3. After body fluid exposure risk	873	833	95.4%	26.836	<0.005
4. After patient contact	1218	1019	83.7%	169.959	<0.005
5. After contact with patient surroundings	752	720	95.7%	3.361	>0.05
6. After removing gloves	1437	1272	88.5%	67.367	<0.005

^a^All indications were recommended by the WHO except after removing gloves

**Fig. 3:**
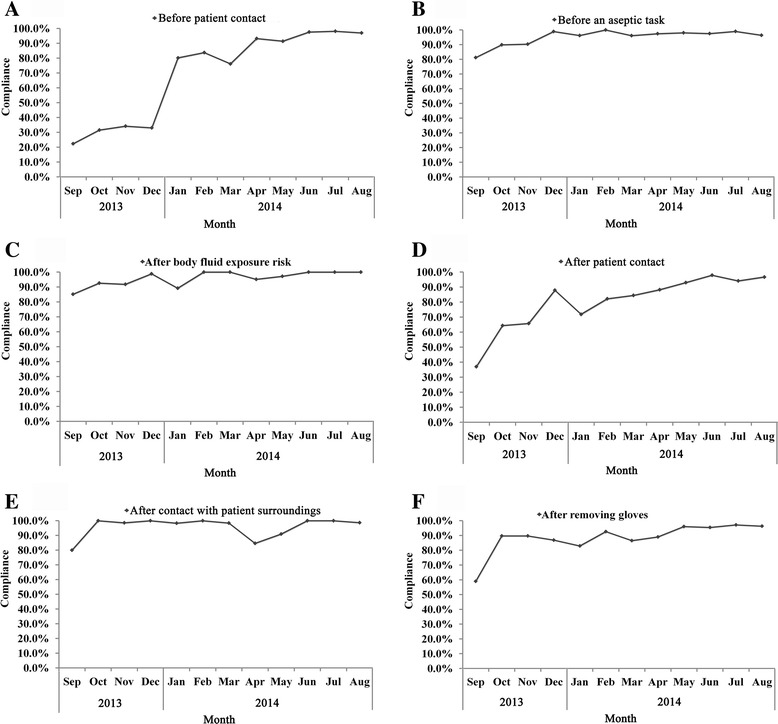
Monthly hand hygiene compliance of before patient contact (**a**), before an aseptic task (**b**), after body fluid exposure risk (**c**), after patient contact (**d**), after contact with patient surroundings (**e**) and after removing gloves (**f**)

Among the six hand hygiene indications, after body fluid exposure risk, before an aseptic task, after contact with patient surroundings and after removing gloves showed higher compliances ranging 87.7–100%, 87.4–100%, 84.6–100% and 82.9–97.2% (Fig. 3), respectively. However, the compliance of indications such as before patient contact and after patient contact were extremely low, only 22.4 and 37.0% of hand hygiene opportunities were detected to perform hand hygiene at the beginning of the QCC program. The most important cause of the difference may attribute to the less attention to hand hygiene in the clinical practice. For this reason, HCWs did not develop the handwashing habit in the initial of QCC program, and showed low compliance with hand hygiene before and after patient contact. On the contrary, the hand hygiene compliance was high as some clinical practices were performed, such as after body fluid exposure risk, before an aseptic task. Hence, interventions such as paying a fine were executed to the noncompliant HCWs, and the compliance of before (and after) patient contact raised to 80.2% (87.9%) in January 2014 (December 2013). The results of Cochran Armitage trend test were shown in Table 1. All these hand hygiene indications exhibited significant linear increases except the indication of after contact with patient surroundings, which the *P* value > 0.05.

## Discussion

Hand hygiene has been identified as the most significant, modifiable cause of HAI, yet hand hygiene compliance is negative in China [14–16]. In our hospital, variety of hand hygiene interventions have been explored in the hospital setting, hand hygiene behavior was surveilled every month and correction reports were released. However, hand hygiene adherence behavior is less than expected among HCWs and the compliance is still a challenge. In 2013, the QCC was explored to improve hand hygiene compliance, and HCWs were encouraged to participate in the management of hand hygiene program. In our study, many of the ideas suggested by QCC members were taken into consideration and multifaceted interventions were applied to improve hand hygiene compliance. For the promotion of hand hygiene, the acceptance and implementation are the most critical. In this QCC program, the full participation of HCWs was essential to implement hand hygiene behavior in their clinical practices. Also, the surveillance-evaluation-feedback-intervention model should be recommended for a high, sustained level of hand hygiene compliance.

According to the management mode of QCC, continuous monitoring is necessary for the sustained improvement of hand hygiene compliance. Hence, the covert observers were invited to record the hand hygiene compliance aperiodically and the data was summarized and evaluated at the circle meetings. Although the hand hygiene compliance expressed a significant increase in the covert observation, the dentists and male HCWs should be noted as they showed low compliance than the nurses and the female respectively. Meanwhile, the fact that the compliances of before patient contact and after patient contact were extremely low also revealed these indications required more monitoring and intervention as necessary. Of note, various hand hygiene compliances in Fig. 2 showed a drop between December 2013 and January 2014. The main reason for our analysis should be the coming Chinese New Year holiday in February 2014. In such case, interference from other work decreased hand hygiene compliance, and HCWs paid less attention to their hand hygiene behaviors in medical practices. Hence, an additional intervention which assuming corresponding responsibility to the circle head was implemented. Consequently, the hand hygiene program is a long, evolutionary process, and improvement of hand hygiene compliance will be difficult to achieve without systemic and multifaceted interventions.

This study was conducted at a major teaching stomatology hospital, and it had several strengths and limitations. The strengths were as follows. First, the QCC theory was used for the improvement of hand hygiene program, and multifaceted strategies were used. Second, covert observers were chosen to record hand hygiene opportunities and actions, and the HCWs were not aware of the covert observers. The advantage of using covert observation can avoid the Hawthorne effect [17, 18] and maintain patient privacy. Third, this study included a very large number of hand hygiene opportunities and actions. Additionally, we analyzed separately by professional category and gender, and significant differences were observed in these parameters. These help to pinpoint areas of strength and weakness in hand hygiene program. However, there were several limitations. One limitation of the method used to measure hand hygiene compliance could lead to selection bias or information bias if the covert observers selected HCWs non-randomly or recorded incorrectly [19, 20]. In anticipation of this bias, we designed a large sample size, and all covert observers were trained and participated in an examination before monitoring hand hygiene behavior. Also, the timing and appropriate technique components of compliance should also be measured and a scale up of this QCC program in other departments is still necessary.

## Conclusions

The improvement of hand hygiene compliance is a worldwide program, and the promotion of hand hygiene behavior needs a long-term effort. QCC can inspire the HCWs to participate in the management of hand hygiene program, systemic and multifaceted interventions will be more effective in improving hand hygiene compliance. Though training is the most basic intervention element, surveillance, evaluation and feedback should be explored as additional interventions to ensure that hand hygiene compliance is achieved and sustained at high levels.

## Additional files

Additional file 1: The basic steps and approximate schedule of quality control circle (QCC) program. (DOC 32 kb)
Additional file 2: The major agenda and interventions proposed in circle meetings during the period of quality control circle (QCC) program. (DOCX 15 kb)
Additional file 3: The process and basic data of Cochran Armitage trend test applied for the linear trend of hand hygiene compliance. (DOCX 29 kb)

## Abbreviations

HAI: Healthcare-associated infection
HCWs: Healthcare workers
PDCA: Plan-do-check-act
QCC: Quality control circle
WHO: World Health Organization
