# Supplementary material for: Role of quality control circle in sustained improvement of hand hygiene compliance: an observational study in a stomatology hospital in Shandong, China
Source: Antimicrob Resist Infect Control. 2016 Dec 8;5:54. doi: 10.1186/s13756-016-0160-1 (PMC5146823; doi:10.1186/s13756-016-0160-1)
Supplement: Additional file 1: — The basic steps and approximate schedule of quality control circle (QCC) program. (DOC 32 kb) [file 13756_2016_160_MOESM1_ESM.doc]

**Additional file 1**

1. theme selection (Sep. 2013)

2. QCC planning (Sep. 2013)

3. Understanding status quo (Sep. 2013)

4. Setting goal (Sep. 2013)

5. Reason analysis (Sep. 2013)

6. Formulating strategy (Sep. 2013)

Plan

Do

Check

Act

7. Execution and evaluation (Oct. 2013)

8. Results confirmation (Jun. 2014)

9. Standardization (Aug. 2014)

10. Review and improvement (Aug. 2014)

Yes

No

**Figure S1** The basic steps and approximate schedule of quality control circle (QCC) program.
