# Supplementary material for: Role of quality control circle in sustained improvement of hand hygiene compliance: an observational study in a stomatology hospital in Shandong, China
Source: Antimicrob Resist Infect Control. 2016 Dec 8;5:54. doi: 10.1186/s13756-016-0160-1 (PMC5146823; doi:10.1186/s13756-016-0160-1)
Supplement: Additional file 2: — The major agenda and interventions proposed in circle meetings during the period of quality control circle (QCC) program. (DOCX 15 kb) [file 13756_2016_160_MOESM2_ESM.docx]

**Additional file 2**

**Table S1** The major agenda and interventions proposed in circle meetings during the period of quality control circle (QCC) program

| **Date** | **Topics** | **Interventions** | **Participants** |
| --- | --- | --- | --- |
| September 4, 2013 | Launched the QCC program and Training the basic information of QCC and PDCA method | - | All staff in department of implant dentistry  Infection control officers |
| September 6, 2013 | Analyzing reasons | - | All QCC members  Infection control officers |
| September 10, 2013 | Formulated intervention measures | 1.Addition of hand hygiene products to each dental unit  2. Establishing a covert observer unit  3. Circle meetings at least once a month  4.Reward and punishment measures | Circle head  Infection control officers |
| September 11, 2013 | Released intervention measures and added hand hygiene products | - | All QCC members  Infection control officers |
| September 13, 2013 | Trained covert observers | - | Infection control officers  Medical students |
| October 8, 2013 | Announced and posting up the compliance | posting up a red flag to the exceptional individuals | All QCC members  Infection control officers |
| November 12, 2013 | Announced and posting up the compliance | posting up a red flag to the exceptional individuals | All QCC members  Infection control officers |
| December 5, 2013 | Announced and posting up the compliance | posting up a red flag to the exceptional individuals | All QCC members  Infection control officers |
| January 6, 2014 | Improved compliance of before (and after) touching a patient | corresponding responsibility to the circle head were implemented | Circle head  Infection control officers |
| February 2014-August 2014 | Announced and posting up the compliance | posting up a red flag to the exceptional individuals and executing a fine to the non compliant members | All QCC members  Infection control officers |
| July 18, 2014 | Developed hand hygiene procedures and surveillance guide | - | Circle head  Infection control officers |
| August 22, 2014 | Revised procedures and standardization | - | Circle head  Infection control officers |
| September 11, 2014 | Announced termination of the QCC program and promoted to the rest departments | - | All QCC members  Infection control officers |

-: no interventions applied.
