# Supplementary material for: Role of quality control circle in sustained improvement of hand hygiene compliance: an observational study in a stomatology hospital in Shandong, China
Source: Antimicrob Resist Infect Control. 2016 Dec 8;5:54. doi: 10.1186/s13756-016-0160-1 (PMC5146823; doi:10.1186/s13756-016-0160-1)
Supplement: Additional file 3: — The process and basic data of Cochran Armitage trend test applied for the linear trend of hand hygiene compliance. (DOCX 29 kb) [file 13756_2016_160_MOESM3_ESM.docx]

**Additional file 3**

**Table S1.** The process and basic data of Cochran Armitage trend test applied for the indication of before patient contact.

| Date | *n* | *t* | Compliance | *Z* | *tZ* | *nZ* | *nZ^2^* |
| --- | --- | --- | --- | --- | --- | --- | --- |
| Sep. 2013 | 76 | 17 | 22.4% | 1 | 17 | 76 | 76 |
| Oct. 2013 | 76 | 24 | 31.6% | 2 | 48 | 152 | 304 |
| Nov. 2013 | 79 | 27 | 34.2% | 3 | 81 | 237 | 711 |
| Dec. 2013 | 115 | 38 | 33.0% | 4 | 152 | 460 | 1840 |
| Jan. 2014 | 116 | 93 | 80.2% | 5 | 465 | 580 | 2900 |
| Feb. 2014 | 80 | 67 | 83.8% | 6 | 402 | 480 | 2880 |
| Mar. 2014 | 88 | 67 | 76.1% | 7 | 469 | 616 | 4312 |
| Apr. 2014 | 161 | 150 | 93.2% | 8 | 1200 | 1288 | 10304 |
| May 2014 | 128 | 117 | 91.4% | 9 | 1053 | 1152 | 10368 |
| Jun. 2014 | 164 | 160 | 97.6% | 10 | 1600 | 1640 | 16400 |
| Jul. 2014 | 161 | 158 | 98.1% | 11 | 1738 | 1771 | 19481 |
| Aug. 2014 | 166 | 161 | 97.0% | 12 | 1932 | 1992 | 23904 |
| Total | 1410 | 1079 | - | - | 9157 | 10444 | 93480 |

*n* = monthly hand hygiene opportunity; *t* = monthly hand hygiene action; *Z* = value of monthly hand hygiene compliance.

**Table S2.** The process and basic data of Cochran Armitage trend test applied for the indication of before an aseptic task.

| Date | *n* | *t* | Compliance | *Z* | *tZ* | *nZ* | *nZ^2^* |
| --- | --- | --- | --- | --- | --- | --- | --- |
| Sep. 2013 | 69 | 56 | 81.2% | 1 | 56 | 69 | 69 |
| Oct. 2013 | 69 | 62 | 89.9% | 2 | 124 | 138 | 276 |
| Nov. 2013 | 72 | 65 | 90.3% | 3 | 195 | 216 | 648 |
| Dec. 2013 | 92 | 91 | 98.9% | 4 | 364 | 368 | 1472 |
| Jan. 2014 | 53 | 51 | 96.2% | 5 | 255 | 265 | 1325 |
| Feb. 2014 | 53 | 53 | 100.0% | 6 | 318 | 318 | 1908 |
| Mar. 2014 | 77 | 74 | 96.1% | 7 | 518 | 539 | 3773 |
| Apr. 2014 | 115 | 112 | 97.4% | 8 | 896 | 920 | 7360 |
| May 2014 | 103 | 101 | 98.1% | 9 | 909 | 927 | 8343 |
| Jun. 2014 | 80 | 78 | 97.5% | 10 | 780 | 800 | 8000 |
| Jul. 2014 | 98 | 97 | 99.0% | 11 | 1067 | 1078 | 11858 |
| Aug. 2014 | 110 | 106 | 96.4% | 12 | 1272 | 1320 | 15840 |
| Total | 991 | 946 | - | - | 6754 | 6958 | 60872 |

*n* = monthly hand hygiene opportunity; *t* = monthly hand hygiene action; *Z* = value of monthly hand hygiene compliance.

**Table S3.** The process and basic data of Cochran Armitage trend test applied for the indication of after body fluid exposure risk.

| Date | *n* | *t* | Compliance | *Z* | *tZ* | *nZ* | *nZ^2^* |
| --- | --- | --- | --- | --- | --- | --- | --- |
| Sep. 2013 | 81 | 69 | 85.2% | 1 | 69 | 81 | 81 |
| Oct. 2013 | 81 | 75 | 92.6% | 2 | 150 | 162 | 324 |
| Nov. 2013 | 85 | 78 | 91.8% | 3 | 234 | 255 | 765 |
| Dec. 2013 | 86 | 85 | 98.8% | 4 | 340 | 344 | 1376 |
| Jan. 2014 | 83 | 74 | 89.2% | 5 | 370 | 415 | 2075 |
| Feb. 2014 | 65 | 65 | 100.0% | 6 | 390 | 390 | 2340 |
| Mar. 2014 | 57 | 57 | 100.0% | 7 | 399 | 399 | 2793 |
| Apr. 2014 | 62 | 59 | 95.2% | 8 | 472 | 496 | 3968 |
| May 2014 | 71 | 69 | 97.2% | 9 | 621 | 639 | 5751 |
| Jun. 2014 | 66 | 66 | 100.0% | 10 | 660 | 660 | 6600 |
| Jul. 2014 | 70 | 70 | 100.0% | 11 | 770 | 770 | 8470 |
| Aug. 2014 | 66 | 66 | 100.0% | 12 | 792 | 792 | 9504 |
| Total | 873 | 833 | - | - | 5267 | 5403 | 44047 |

*n* = monthly hand hygiene opportunity; *t* = monthly hand hygiene action; *Z* = value of monthly hand hygiene compliance.

**Table S4.** The process and basic data of Cochran Armitage trend test applied for the indication of after patient contact.

| Date | *n* | *t* | Compliance | *Z* | *tZ* | *nZ* | *nZ^2^* |
| --- | --- | --- | --- | --- | --- | --- | --- |
| Sep. 2013 | 73 | 27 | 37.0% | 1 | 27 | 73 | 73 |
| Oct. 2013 | 73 | 47 | 64.4% | 2 | 94 | 146 | 292 |
| Nov. 2013 | 76 | 50 | 65.8% | 3 | 150 | 228 | 684 |
| Dec. 2013 | 99 | 87 | 87.9% | 4 | 348 | 396 | 1584 |
| Jan. 2014 | 103 | 74 | 71.8% | 5 | 370 | 515 | 2575 |
| Feb. 2014 | 56 | 46 | 82.1% | 6 | 276 | 336 | 2016 |
| Mar. 2014 | 90 | 76 | 84.4% | 7 | 532 | 630 | 4410 |
| Apr. 2014 | 93 | 82 | 88.2% | 8 | 656 | 744 | 5952 |
| May 2014 | 128 | 119 | 93.0% | 9 | 1071 | 1152 | 10368 |
| Jun. 2014 | 142 | 139 | 97.9% | 10 | 1390 | 1420 | 14200 |
| Jul. 2014 | 135 | 127 | 94.1% | 11 | 1397 | 1485 | 16335 |
| Aug. 2014 | 150 | 145 | 96.7% | 12 | 1740 | 1800 | 21600 |
| Total | 1218 | 1019 | - | - | 8051 | 8925 | 80089 |

*n* = monthly hand hygiene opportunity; *t* = monthly hand hygiene action; *Z* = value of monthly hand hygiene compliance.

**Table S5.** The process and basic data of Cochran Armitage trend test applied for the indication of after contact with patient surroundings.

| Date | *n* | *t* | Compliance | *Z* | *tZ* | *nZ* | *nZ^2^* |
| --- | --- | --- | --- | --- | --- | --- | --- |
| Sep. 2013 | 60 | 48 | 80.0% | 1 | 48 | 60 | 60 |
| Oct. 2013 | 60 | 60 | 100.0% | 2 | 120 | 120 | 240 |
| Nov. 2013 | 68 | 67 | 98.5% | 3 | 201 | 204 | 612 |
| Dec. 2013 | 63 | 63 | 100.0% | 4 | 252 | 252 | 1008 |
| Jan. 2014 | 58 | 57 | 98.3% | 5 | 285 | 290 | 1450 |
| Feb. 2014 | 73 | 73 | 100.0% | 6 | 438 | 438 | 2628 |
| Mar. 2014 | 63 | 62 | 98.4% | 7 | 434 | 441 | 3087 |
| Apr. 2014 | 65 | 55 | 84.6% | 8 | 440 | 520 | 4160 |
| May 2014 | 66 | 60 | 90.9% | 9 | 540 | 594 | 5346 |
| Jun. 2014 | 42 | 42 | 100.0% | 10 | 420 | 420 | 4200 |
| Jul. 2014 | 58 | 58 | 100.0% | 11 | 638 | 638 | 7018 |
| Aug. 2014 | 76 | 75 | 98.7% | 12 | 900 | 912 | 10944 |
| Total | 752 | 720 | - | - | 4716 | 4889 | 40753 |

*n* = monthly hand hygiene opportunity; *t* = monthly hand hygiene action; *Z* = value of monthly hand hygiene compliance.

**Table S6.** The process and basic data of Cochran Armitage trend test applied for the indication of after removing gloves.

| Date | *n* | *t* | Compliance | *Z* | *tZ* | *nZ* | *nZ^2^* |
| --- | --- | --- | --- | --- | --- | --- | --- |
| Sep. 2013 | 117 | 69 | 59.0% | 1 | 69 | 117 | 117 |
| Oct. 2013 | 117 | 105 | 89.7% | 2 | 210 | 234 | 468 |
| Nov. 2013 | 117 | 105 | 89.7% | 3 | 315 | 351 | 1053 |
| Dec. 2013 | 130 | 113 | 86.9% | 4 | 452 | 520 | 2080 |
| Jan. 2014 | 117 | 97 | 82.9% | 5 | 485 | 585 | 2925 |
| Feb. 2014 | 109 | 101 | 92.7% | 6 | 606 | 654 | 3924 |
| Mar. 2014 | 119 | 103 | 86.6% | 7 | 721 | 833 | 5831 |
| Apr. 2014 | 128 | 114 | 89.1% | 8 | 912 | 1024 | 8192 |
| May 2014 | 128 | 123 | 96.1% | 9 | 1107 | 1152 | 10368 |
| Jun. 2014 | 110 | 105 | 95.5% | 10 | 1050 | 1100 | 11000 |
| Jul. 2014 | 108 | 105 | 97.2% | 11 | 1155 | 1188 | 13068 |
| Aug. 2014 | 137 | 132 | 96.4% | 12 | 1584 | 1644 | 19728 |
| Total | 1437 | 1272 | - | - | 8666 | 9402 | 78754 |

*n* = monthly hand hygiene opportunity; *t* = monthly hand hygiene action; *Z* = value of monthly hand hygiene compliance.

**Table S7.** The process and basic data of Cochran Armitage trend test applied for the overall hand hygiene compliance.

| Date | *n* | *t* | Compliance | *Z* | *tZ* | *nZ* | *nZ^2^* |
| --- | --- | --- | --- | --- | --- | --- | --- |
| Sep. 2013 | 476 | 286 | 60.1% | 1 | 286 | 476 | 476 |
| Oct. 2013 | 476 | 373 | 78.4% | 2 | 746 | 952 | 1904 |
| Nov. 2013 | 497 | 392 | 78.9% | 3 | 1176 | 1491 | 4473 |
| Dec. 2013 | 585 | 477 | 81.5% | 4 | 1908 | 2340 | 9360 |
| Jan. 2014 | 530 | 446 | 84.2% | 5 | 2230 | 2650 | 13250 |
| Feb. 2014 | 436 | 405 | 92.9% | 6 | 2430 | 2616 | 15696 |
| Mar. 2014 | 494 | 439 | 88.9% | 7 | 3073 | 3458 | 24206 |
| Apr. 2014 | 624 | 572 | 91.7% | 8 | 4576 | 4992 | 39936 |
| May 2014 | 624 | 589 | 94.4% | 9 | 5301 | 5616 | 50544 |
| Jun. 2014 | 604 | 590 | 97.7% | 10 | 5900 | 6040 | 60400 |
| Jul. 2014 | 630 | 615 | 97.6% | 11 | 6765 | 6930 | 76230 |
| Aug. 2014 | 705 | 685 | 97.2% | 12 | 8220 | 8460 | 101520 |
| Total | 6681 | 5869 | - | - | 42611 | 46021 | 397995 |

*n* = monthly hand hygiene opportunity; *t* = monthly hand hygiene action; *Z* = value of monthly hand hygiene compliance.
